# Big Data, Natural Language Processing, and Deep Learning to Detect and Characterize Illicit COVID-19 Product Sales: Infoveillance Study on Twitter and Instagram

**DOI:** 10.2196/20794

**Published:** 2020-08-25

**Authors:** Tim Ken Mackey, Jiawei Li, Vidya Purushothaman, Matthew Nali, Neal Shah, Cortni Bardier, Mingxiang Cai, Bryan Liang

**Affiliations:** 1 Department of Anesthesiology and Division of Infectious Diseases and Global Public Health School of Medicine University of California, San Diego La Jolla, CA United States; 2 Department of Healthcare Research and Policy University of California, San Diego La Jolla, CA United States; 3 Global Health Policy and Data Institute San Diego, CA United States; 4 S-3 Research LLC San Diego, CA United States; 5 Masters in Public Health Program Department of Family Medicine and Public Health University of California, San Diego - School of Medicine La Jolla, CA United States; 6 Masters Program in Global Health Department of Anthropology University of California, San Diego La Jolla, CA United States

**Keywords:** COVID-19, coronavirus, infectious disease, social media, surveillance, infoveillance, infodemiology, infodemic, fraud, cybercrime

## Abstract

**Background:**

The coronavirus disease (COVID-19) pandemic is perhaps the greatest global health challenge of the last century. Accompanying this pandemic is a parallel “infodemic,” including the online marketing and sale of unapproved, illegal, and counterfeit COVID-19 health products including testing kits, treatments, and other questionable “cures.” Enabling the proliferation of this content is the growing ubiquity of internet-based technologies, including popular social media platforms that now have billions of global users.

**Objective:**

This study aims to collect, analyze, identify, and enable reporting of suspected fake, counterfeit, and unapproved COVID-19–related health care products from Twitter and Instagram.

**Methods:**

This study is conducted in two phases beginning with the collection of COVID-19–related Twitter and Instagram posts using a combination of web scraping on Instagram and filtering the public streaming Twitter application programming interface for keywords associated with suspect marketing and sale of COVID-19 products. The second phase involved data analysis using natural language processing (NLP) and deep learning to identify potential sellers that were then manually annotated for characteristics of interest. We also visualized illegal selling posts on a customized data dashboard to enable public health intelligence.

**Results:**

We collected a total of 6,029,323 tweets and 204,597 Instagram posts filtered for terms associated with suspect marketing and sale of COVID-19 health products from March to April for Twitter and February to May for Instagram. After applying our NLP and deep learning approaches, we identified 1271 tweets and 596 Instagram posts associated with questionable sales of COVID-19–related products. Generally, product introduction came in two waves, with the first consisting of questionable immunity-boosting treatments and a second involving suspect testing kits. We also detected a low volume of pharmaceuticals that have not been approved for COVID-19 treatment. Other major themes detected included products offered in different languages, various claims of product credibility, completely unsubstantiated products, unapproved testing modalities, and different payment and seller contact methods.

**Conclusions:**

Results from this study provide initial insight into one front of the “infodemic” fight against COVID-19 by characterizing what types of health products, selling claims, and types of sellers were active on two popular social media platforms at earlier stages of the pandemic. This cybercrime challenge is likely to continue as the pandemic progresses and more people seek access to COVID-19 testing and treatment. This data intelligence can help public health agencies, regulatory authorities, legitimate manufacturers, and technology platforms better remove and prevent this content from harming the public.

## Introduction

The novel coronavirus (2019-nCoV; also known as severe acute respiratory syndrome coronavirus 2 [SARS-Cov-2]) and associated diagnosis, the coronavirus disease (COVID-19), has created a global crisis. Its broad effect has not been seen since the days of the 1918 influenza pandemic that impacted 200-700 million people (1/3 of the world’s population at the time) and resulted in global mortality of 50-100 million [1]. Impacting virtually every corner of the world after initially appearing in Wuhan, China, COVID-19’s threat to humanity is broad [2]. Measures to fight the threat, including social distancing, quarantine, and limited commercial activity, are now the global norm, along with travel restrictions and other measures put into place in an effort to contain the pandemic [3]. 

With the advent of social media, an information-sharing culture, and technological dispersion throughout the world to access these platforms (ie, mobile, broadband access) the more than 2.9 billion global social media users now have more information resources to help them understand and protect themselves against the coronavirus [4]. Indeed, social media platforms represent one of the most accessible sources of health information and are now being used by agencies such as the World Health Organization (WHO), US Centers for Disease Control and Prevention, US Food and Drug Administration (FDA), and others [5,6]. Social media conversations are also important for understanding public sentiment, user behavior, and disease transmission dynamics during outbreaks. For example, Twitter has been used extensively for “infoveillance” approaches to assess past outbreaks such as H1N1, Zika virus, and the Ebola outbreak [7-10].

Yet accompanying the strong utility of internet technologies and social media to positively impact outbreak response and communication is a nefarious underpinning: a criminal element that is now across and within social media seeking to capitalize on confusion, fears, and the acute needs of the public. Labeled by the WHO as an “infodemic,” where there is an overabundance of information, some of which includes misinformation and enables COVID-19–related cybercrime, this parallel information epidemic is now a serious challenge to ensuring the success of public health objectives of mitigating the spread of COVID-19 [11,12]. Beyond misinformation about the etiology and basic facts of COVID-19, which the WHO is trying to counter with its COVID-19 “Myth Busters” website, other forms of COVID-19–related cybercrime are now widespread [13].

Documented COVID-19–related cybercrimes include fake coronavirus applications that are actually malware, phishing scams using email, text message campaigns and robocalls, economic scams regarding government assistance or relief, and a host of suspect and counterfeit COVID-19 products now sold online [14,15]. Numerous news outlets have reported the use of online platforms including popular social media sites as a source for suspect COVID-19–related health products [16]. For example, COVID-19 “cures” have appeared across major electronic commerce (e-commerce) sites including Amazon.com (which reported removing a million fake COVID-19 product listings), Shopify store vendors, and other reselling and auction platforms such as eBay [17,18]. Unapproved COVID-19 test kits, both serologic as well as reverse transcriptase polymerase chain reaction tests, are being sold by multiple sources including Twitter, Instagram, and Reddit [19]. Finally, the dark web has been identified as a source for counterfeit COVID-19 therapeutics, including biological products such as blood plasma [20].

Hence, there is significant need to assess the characteristics of illegal online COVID-19 product marketing and sale at different stages of the pandemic. In response, this study used big data, natural language processing (NLP), and machine learning to identify the marketing and sale of suspect and unapproved COVID-19 cures, testing kits, and other questionable treatments at earlier stages of the pandemic on two popular social media platforms: Twitter and Instagram. We also describe an approach to visualize findings in a customized data dashboard to enable public health intelligence and reporting to authorities. 

## Methods

### Overview

**Figure 1:**
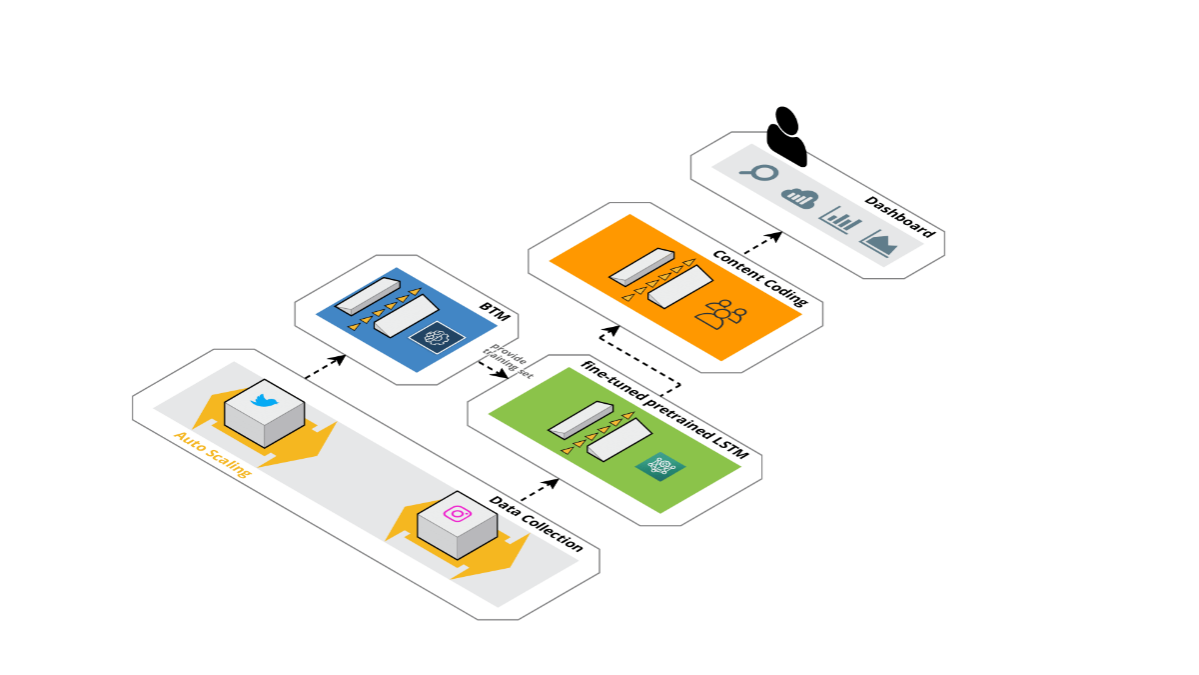
Summary of study methodology. The first phase (yellow) is collection of data from the public streaming Twitter application programming interface and using a web scraper on Instagram to collect social media posts filtered for COVID-19–related keywords; the second phase (blue) used BTM to isolate topic clusters related to COVID-19 product sales to develop an initial training set for classification of posts using a deep learning algorithm (green). Data output by the deep learning classifier was then manually coded for true signals and selling characteristics (orange). Finally, the visualization of labeled data on a customized dashboard to enable public health intelligence and reporting to public health agencies was conducted (grey). BTM: biterm topic model; COVID-19: coronavirus disease; LSTM: long short-term memory.

This retrospective big data study was conducted in two phases: (1) data collection using the public streaming Twitter application programming interface (API) and the use of web scraping on Instagram to collect social media posts filtered for COVID-19–related keywords and (2) data analysis using NLP to isolate topic clusters related to COVID-19 product sales combined with a deep learning algorithm to classify a larger volume of social media posts for classification of “signal” posts (ie, posts confirmed as associated with COVID-19 product marketing and selling; see Figure 1 for summary). Data storage and analysis was conducted on an on-premise deep learning workstation in combination with a series of virtual machines deployed on Amazon Web Service cloud-computing. Additional details of the data collection, processing, and analysis are available in Multimedia Appendix 1.

### Data Collection

This study first applied a systematic approach to conduct data mining on Twitter by filtering the public streaming API for keywords associated with COVID-19 to collect a large corpus of general COVID-19–related conversations from March 3 to April 11, 2020. The same set of keywords were used to collect data from Instagram using a web scraper built in the programming language Python. We identified general COVID-19–related keywords based on manual searches on each of the platforms, which included different iterations of “COVID-19” (eg, “covid19,” “corona,” “coronavirus,” “coronavid19”), with these keywords converted into hashtags to conduct searches on Instagram. Text of tweets and Instagram posts were captured, as well as retweets and other metadata including likes; favorites; comments; replies; use of similar hashtags; and associated media, hyperlinks, and metadata of posts (eg, time stamp, geolocation, and account information). This metadata was primarily used to identify any potential temporal trends associated with selling posts, account characteristics of sellers, interaction of posts with other users, geospatial information, and to characterize hyperlinks to external websites that were imbedded in selling posts.

After collecting an initial corpus of tweets and Instagram posts using general COVID-19 keywords, we then filtered the corpus for additional terms we believed to be associated with the marketing and sale of illegal, suspect, counterfeit, and otherwise misleading COVID-19–related products and treatments as first identified in manual searches. A full list of all filtered terms used in this study is available in Multimedia Appendix 1.

### Data Analysis Using Unsupervised and Supervised Machine Learning Approaches

After collecting Twitter and Instagram posts, and then filtering for illegal marketing and sales terms, we processed the data by removing hashtags and stop words prior to textual analysis. To our knowledge, there is no existing training set related to detecting suspect COVID-19 products in the context of the current pandemic. This necessitated using a combination of unsupervised and supervised machine learning approaches to detect an initial training set of “signal” posts from each platform that were then used to train a supervised machine learning classifier using a deep learning model.

We first used an “unsupervised” NLP approach to group and summarize all the content of filtered social media data stratified by different product groups of filtered terms. This was accomplished by assessing the entire corpora of COVID-19 filtered data using the biterm topic model (BTM) to both identify initial signal posts in the absence of labelled data and to curate an initial labelled training set for supervised machine learning purposes (see Multimedia Appendix 1 for additional details). We have used BTM in prior published studies to detect social media conversations related to substance use behavior, illicit drug diversion, online wildlife trafficking, and corruption-related activities [21-23].

Signal posts detected in our BTM phase were then used as our training set for a deep learning classifier designed to conduct supervised classification on the entire corpus of filtered social media posts. For this study, we adopted an existing deep learning model used to detect online controlled substance and illicit drug sales as previously published by authors [24]. Although the original deep learning model was trained on social media posts labelled for illegal online drug sales, the signal texts of these two data sets contained very similar features (eg, specific “seller information” and “product information” features). Hence, the pretrained model helped us detect these specific “selling” features targeted for COVID-19 sellers and products. This was due to the fact that our corpus of social media posts was already purposely filtered for COVID-19 keywords (ie, not illicit drug-related terms).

Hence, this combination of unsupervised and supervised machine learning approaches enabled us to quickly develop a data collection and analysis approach for an emerging infoveillance challenge given the rapidity and large volume of COVID-19–related data and the evolving nature of the pandemic itself.

### Content Coding

After classification by our deep learning algorithm, posts that were output by the model and classified as possible “signal” were then manually annotated to confirm if they were associated with illegal marketing and sales of COVID-19 health-related products (see Multimedia Appendix 2 for coding scheme details). First, coders independently used a binary coding approach (ie, signal vs nonsignal) to verify if posts included the sale of a COVID-19 health product and if a contact or purchase method was made available. The purpose of this binary coding scheme was to eliminate “noise” in the data set, including COVID-19 news, regulatory product announcements, user discussions about treatments and testing, and legitimate warnings from public health, law enforcement, and other sources about COVID-19 fraud and cybercrime that were not related to product marketing or sales.

Second, we classified signal posts based on what specific COVID-19 product was being offered individually or concurrently (eg, testing kits, protective equipment, masks, and pharmaceuticals). We also conducted content analysis to characterize strategies used to market and sell products using an open inductive coding scheme based on previous work characterizing online drug sellers [22,24-27]. These characteristics included the method of contacting seller, method of payment (if reported), purported modality of order or purchase, and availability of hyperlinks to other internet sources enabling sale.

Coders individually selected parent topic classifications, removed duplicate topics, and evaluated thematic concurrence by independently coding the entire sample of output posts from our machine learning phase. The third, fourth, fifth, and sixth authors coded posts independently and achieved high intercoder reliability (κ=0.92). In case of inconsistent results, authors reviewed and conferred on the correct classification with the first and last authors who have previously published on the subject. 

### Availability of Data and Materials

Data collected on social media platforms is available on request from authors, subject to appropriate deidentification.

### Ethics Approval and Consent to Participate

Ethics approval and consent to participate was not required for this study. All information collected from this study was from the public domain, and the study did not involve any interaction with users. Indefinable user information was removed from the study results.

## Results

### Collected Data

Data was collected from March 3 to April 11, 2020, via the Twitter public API stream and from February 5 to May 7 via the web scrapper built for Instagram. During this period, we collected a total of 6,029,323 tweets and 204,597 Instagram posts that included a COVID-19 general term and that were also filtered for terms associated with suspect marketing and sale of COVID-19 products. After using our deep learning algorithm to classify all posts filtered for marketing and sales terms, we manually annotated and confirmed 1271 tweets of which 1042 were unique (see Textbox 1 for Twitter examples) and 596 Instagram posts (see Textbox 2 for Instagram examples) associated with questionable sales of COVID-19–related products.

**Product categories and example signal posts for suspect coronavirus disease–related products on Twitter.**

**Immunity boosting kits**

“****** is safe for the whole family. Support your immune system with #****** at app.elify.com/vbc/6pf3pvak44…

#COVID-19 #coronavirus #FluSeason #ImmuneSystem #immunebooster #hydrosolsilver #antiviral #antivirus” - March 16, 2020 @******

**Coronavirus disease (COVID-19) testing kit**

“Negative Test Results Product: Fast SARS-CoV-2 Detection Igm/IgG Bioassay disposable one time use kit 4 minute screening.” - March 25, 2020 @******

**COVID-19 IgG/IgM antibody detection kits**

“SARS-CoV-2 (COVID-19) IgM / IgG Antibody Fast Detection Kit (Colloidal Gold) New Coronavirus IgM / IgG Antibody Rapid Detection Kit (colloidal gold method)” - March 21, 2020 @******

**Personal protection equipment (PPE)/masks/gloves**

“10/50/100pcs Antiviral Disposable Face Mask Anti Dust Anti Influenza Face Mouth Mask For Coronavirus Clear Viruses Tool dropship kawaicorner.com/product/10-50-...” - March 13, 2020 @******

**Alleged COVID-19 cures**

“#COVID19 #CoronaOutbreak #Coronavirustexas #Coronachina #ChinaCoronaVirus #coronavirusnigeria found out COVID19 can be cured by the mixture of salivary water extracted from plantain stem, pawpaw tree, scent leaf and Garlic. @****** @WHO @Fmohnigeria @realDonaldTrump @ChinaDaily” - March 2, 2020 @******

**Multiple products (PPE, testing kits, etc)**

“All available. #COVID19 RNA Preservation Kit (with Swab) COVID-19 IgG/IgM Rapid Test Cassette #Disposable protection suits Infrared forehead thermometer. Disposable Protective mask Pls contact me:

Whatsapp: ******

Email: ******” - March 23, 2020 @******

**Product categories and example signal posts for suspect coronavirus disease–related products on Instagram.**

**Immunity boosting kits**

“Happy Monday! ****** is a perfect immune boost for winter illnesses. Get yours before stock runs out.

 #killgerms #germs #nhs #coronavirus #covid #covid2019 #vitaminc #zinc #lambertshealthcare #antibacterial #hillcrestpharmacy #hollandpark #nottinghill #w11”

**Coronavirus disease (COVID-19) testing kit**

“Antibody Rapid Test Coronavirus (COVID-19) ******

Test in 4 Easy Steps

FAST Testing time: 15 min

Accuracy: 92%

Certifications: CE.

Package content = 100 Units

For quantity discounts Please call : ******

 #covid #covidtest #coronavirus #rapidtest #covidtestkit #coronatest #covidrapid #workingwear #protectionwear #jumpsuit #hazmat #hazmatsuit #covidrecovery #covidrapidtest #apd #protection #rapidtestkit #rapidtestcorona #rapidtestcovid #rapidtestkits #healthcare #safety #a #s #covidtesting #coronavir #sifsof”

**COVID-19 IgG/IgM antibody detection kits**

“coronavirus IgM/IgG Test Kit.

1. One box of 25 kits, one box of $192
2. Mode of transport ups plan express. About 3-7 days arrived all over the world. 
3. Division I provide certificate and provide clear customs clearance, tax paying. All you have to do is give us the receiving address. 
4. The delivery time is 3-5 days.
5. Support 100% payment method: PayPal Western Union or Telegraphic. #covid_19 #testkits #corona #coronavirus #covid19”

**Personal protection equipment (PPE)/masks/gloves**

“All of these PPE materials are available, welcome to contact !#facemasks #KN95masks #ffp2mask #sanitizers #gloves #testkit #protectiveclothing #temperaturegun”

**Multiple products (PPE, testing kits, etc)**

“Disposable surgical face mask

****** for coronavirus vaccines and tablets available at very affordable prices hand sanitizer thermometers test kits also available just inbox for your order

#testkit #facemask #thermometer #foreheadthermometer #n95 #3m #8210 #1860 #3plyfacemask #3plymask #/3ply #surgicalfacemask #coronavirus #coronacure #coronavaccine #”

**Figure 2:**
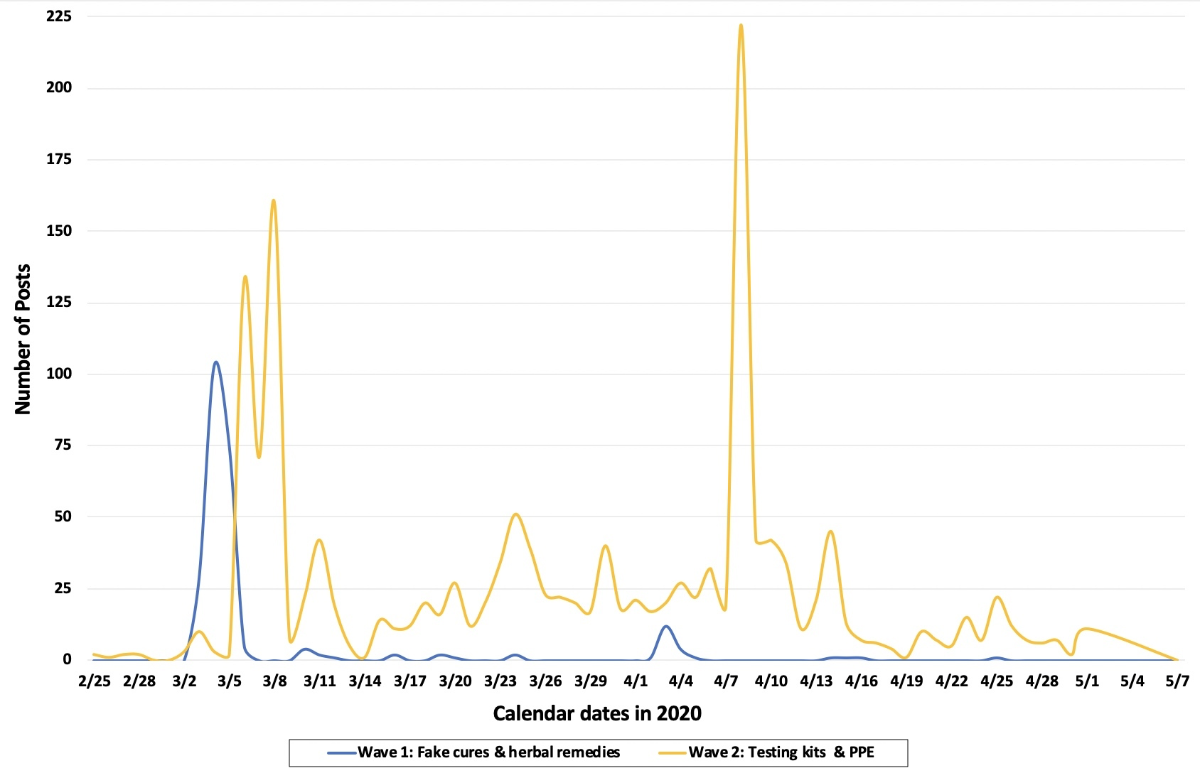
Timeline for volume and topics of signal posts related to suspect coronavirus disease products on Twitter and Instagram. PPE: personal protective equipment.

Based on the periods of data collection and terms used, we generally observed that there was a first spike or “wave” of social media posts related to fake cures and unproven treatments including home remedies, traditional medicines, supplements, essential oils, and other unproven products. This was followed by a second and much larger wave of posts, including offers for sale of suspect COVID-19 testing, screening, and diagnostic products (see Figure 2 for timeline). Hence, we observed that the volume of suspect COVID-19 products on Twitter and Instagram appeared to materialize in two distinct infodemic waves during this relatively early period of the pandemic, with the volume of topics changing over time as news, misinformation, and rumors regarding potential COVID-19 treatments, supplies, testing availability, and other conversations evolved (see Table 1).

**Table 1:** Twitter and Instagram posts associated with questionable sales of COVID-19–related products.

Infodemic wave and COVID-19^a^–related product		Posts, n	
		Twitter (n=1271)^b^	Instagram (n=596)^b^
**Wave 1**			
	Fake cures	209	0
	Herbal medicines	33	6
**Wave 2**			
	Testing kits and PPE^c^	1028	571
	Testing kits	970	410
	PPE	112	181
**Wave 3**			
	Pharmaceuticals	5	22

^a^COVID-19: coronavirus disease.

^b^Total does not add up to sum of posts in waves due to some posts having co-occurring COVID-19–related products.

^c^PPE: personal protective equipment.

The first infodemic wave involved posts related to a variety of unproven treatments (eg, including posts with terms such as “antiviral,” “antibiotic,” and products claiming “immunity boosting” benefits) along with products that were subject to regulatory warnings by the FDA (eg, silver colloidal and chlorine). During this time period of observed fake cures and unproven treatments, news events including claims by InfoWars founder Alex Jones and televangelist Jim Bakker that colloidal silver could treat COVID-19 were followed by regulatory warnings by the FDA, likely leading to increased interest and selling activity on social media for similar products. Other similar rumors regarding preventative measures and COVID-19 treatments were also circulating on the internet and social media at the time [28].

The second wave included terms and posts primarily selling COVID-19 testing kits (eg, terms included “IgM/IgG,” “rapidtest,” and “detectionkit”) in combination with other supplies (eg, masks, protective personal equipment, gloves, and miscellaneous protective gear). During this second wave we observed two distinct spikes of increased volume of posts on or around March 5-10 and April 7-10, 2020. The first spike in March coincided with widespread news coverage about increasing numbers of COVID-19 cases; discussion from state governments about where to get access to testing; press releases from companies discussing development of testing services, such as Quest Diagnostics announcement on March 5, 2020, about new testing services it was developing; and news about testing products undergoing evaluation by the FDA (including under emergency use authorization). The second peak in April coincided with news about testing sites opening and expanding, concerns about a US nationwide shortage of testing capacity, and possible underreporting due to testing backlogs.

Finally, we analyzed the data set for terms associated with promising therapeutics that at the time were announced as possible off-label treatments or were undergoing testing and clinical validation. This included the drugs hydroxychloroquine, chloroquine, remdesivir (proprietary name Veklury, Gilead Sciences), favipiravir (proprietary names Avigan, Abigan, FabiFlu), lopinavir/ritonavir (proprietary name Aluvia, Kaletra, AbbVie Inc), that collectively represent a mix of both proprietary and nonproprietary pharmaceutical treatments, including those that had already been approved by the FDA for non–COVID-19 indications (eg, hydroxychloroquine is approved by the FDA to treat malaria and lupus) and those that are experimental and unapproved drugs. Though we detected some posts in this category, the volume was low relative to waves 1 and 2.

### COVID-19 Product Characteristics

**Figure 3:**
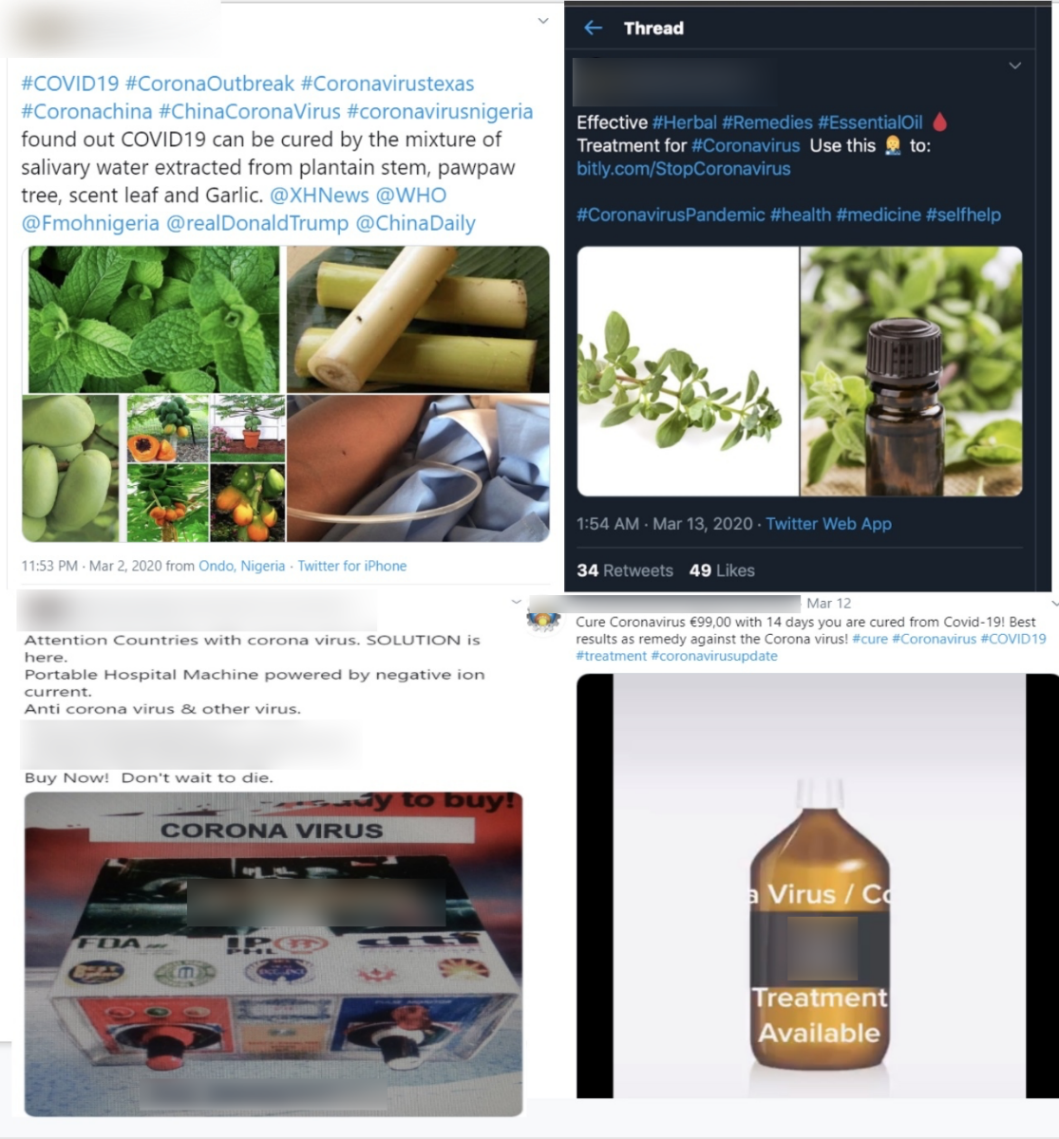
Twitter and Instagram posts related to suspected COVID-19 treatments and remedies. COVID-19: coronavirus disease; WHO: World Health Organization.

In the first wave, which was detected in the earliest stages of the study period from March 3 to April 4, 2020, 242 tweets and 6 Instagram posts (248/1867, 13.28% of all signal posts) advertised the sale of or promoted the use of immune-boosting COVID-19 prevention and treatment products. Herbal products included three general categories: (1) premade herbal or nontraditional remedies; (2) instructions on how to create herbal concoctions and cocktails with purported immunoprotective benefits specific to COVID-19; (3) and other posts including dietary supplements and food products claiming to prevent COVID-19, such as colloidal silver. Other highly questionable products that did not fit into a specific category included a “portable hospital” device that claimed to use a negative ion current to treat COVID-19 and other viruses (see Figure 3 for screenshots).

Premade herbal remedies included products represented as traditional herbal Eastern medicines and compounds but also included consumer items such as lavender spray, pawpaw trees, xylitol, and cow dung with claims of immunoprotective benefits for COVID-19. Sellers of herbal remedies tended to market themselves as doctors or healers with specific reference to Ayurvedic, Eastern, or nontraditional medicine. The descriptive text in some of these posts had misleading claims that combinations of herbal remedies could cure the virus. Moreover, other posts claimed that consumption or proximity to garlic or lomatium could treat COVID-19. Some of the posts used misleading marketing claims such as “approved” or “authorized,” despite these products having no known formal approval for COVID-19 uses. 

**Figure 4:**
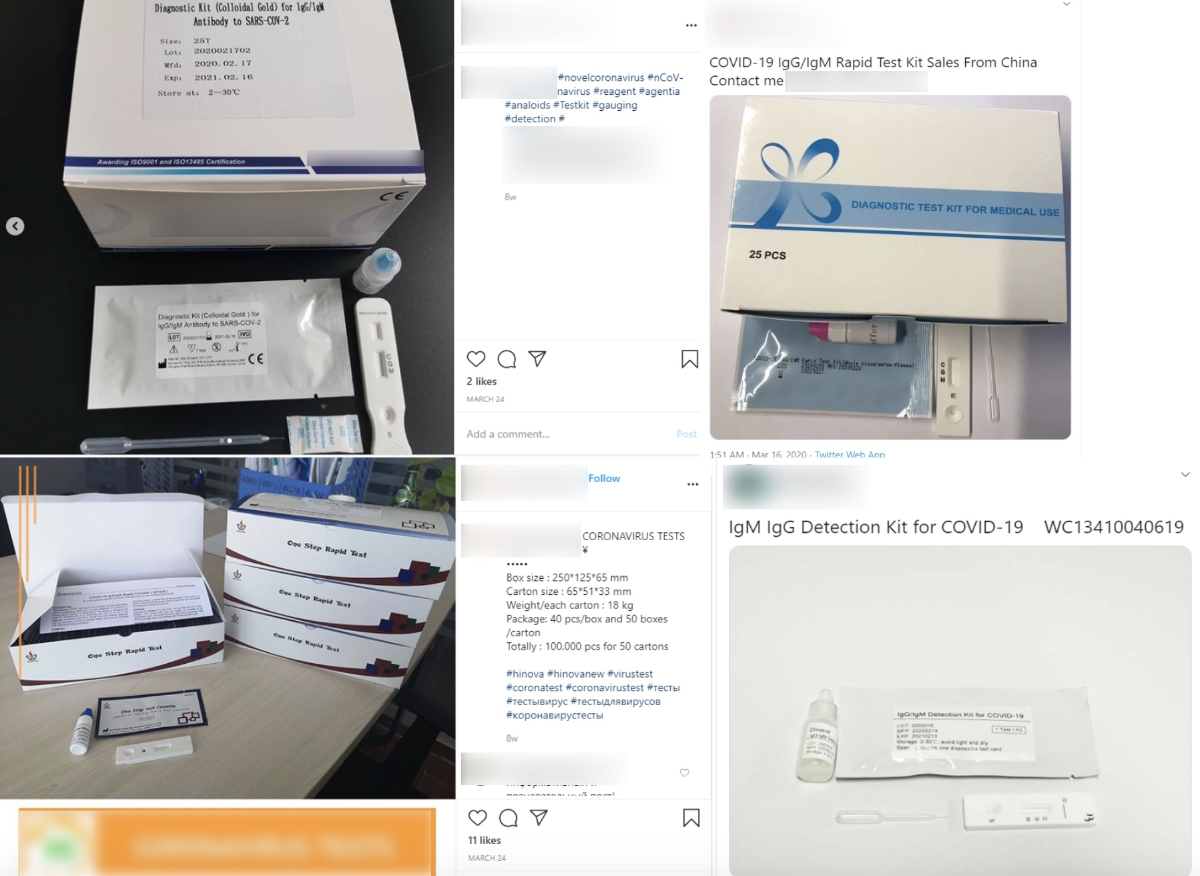
Twitter and Instagram posts related to suspected COVID-19 testing kits. COVID-19: coronavirus disease; SARS-CoV-2: severe acute respiratory syndrome coronavirus 2.

The second wave included the majority of signal posts detected in this study (1028 tweets and 571 Instagram posts, 73.86%) involving the marketing, sale, and distribution of unapproved COVID-19 testing kits (see Figure 4) and were detected from March 6 to April 10, 2020. Most of these posts advertised their testing products as IgM/IgG tests, generally a type of test that detects fluctuating antibody concentrations to determine the presence or absence of SARS-CoV-2. These products were mainly advertised as “rapid test” kits or testing supplies containing colloidal gold. Though there are official commercial rapid lab-based tests to detect IgM/IgG antibodies, in the United States, none are authorized to be sold direct-to-consumer. 

An additional category of testing kit posts included products purportedly approved as at-home kits or “DIY.” However, it should be noted that as of April 21, 2020, only one home testing kit had been approved by the FDA, a home sample collection kit named Pixel by LabCorp (Laboratory Corporation of America). The Pixel kit is only for sample collection at home and the swab samples must be sent to LabCorp processing centers to process COVID-19 results. Other examples of questionable products included those that claimed they could detect COVID-19 by using a fingerstick test or through saliva and urine. Some of the rapid testing kit posts detected in this study also alleged COVID-19 results within minutes using at-home testing and even included questionable claims about the percent accuracy of their tests.

Overall, social media posts involving suspect COVID-19 testing products exhibited similar and identifiable patterns including a picture and description of the specific type of COVID-19 test, the contact information of how to purchase the test kits, and pricing information. Many posts included a claim and mark for a “CE marking,” which is a certification mark that a product conforms with applicable health, safety, and environmental protection standards for the European Economic Area but does not mean the product has been approved by regulatory authorities for COVID-19 screening or diagnosis. Some posts also included users claiming to sell FDA-approved COVID-19 testing equipment (with some products that included spurious FDA labelling in images). Pictures of specific COVID-19 testing kits included variations of the labeled box and materials of the testing kit itself, stock photos of a testing kit, or testing kit packaging. For some posts, the labeling on purported testing kits were written in different languages. Additionally, sellers advertised bundled packages that included COVID-19 tests offered concurrently with personal protective equipment (PPE). 

Specific to PPE, we detected 535 out of 1867 (28.66%) posts that offered the sale of masks, gloves, and other protective gear in conjunction with tests. Posts mentioned sales available via individual purchases, wholesale, or in bundles with equipment such as temperature gauges, protective suits, hand sanitizers, and immunity boosting kits. Additionally, compounds such as silver hydrosol, colloidal silver, and antimicrobial copper were advertised as medical supplies that could confer immune boosting benefits and help with COVID-19 prevention in a variety of ways. PPE and supply posts often included the cost and approximate shipping time, with some linked to an external medical supply company e-commerce site.

**Figure 5:**
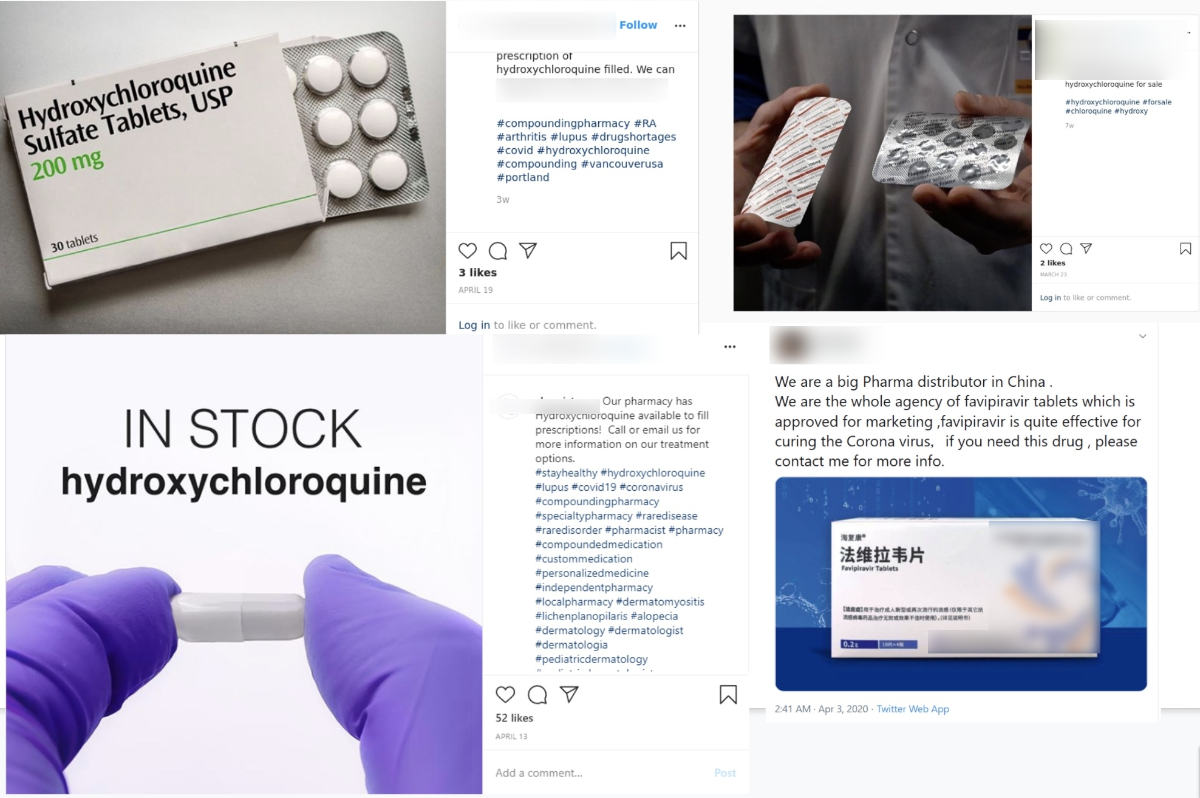
Twitter and Instagram posts related to suspected COVID-19 pharmaceutical drugs. COVID-19: coronavirus disease.

Finally, we detected a small volume of posts offering the sale of COVID-19–related therapeutics, none that, at the time, had been approved for the treatment of COVID-19 (see Figure 5), which were detected from March 5, 2020, to April 13, 2020. The majority of posts reviewed for these therapeutic-related filtered terms contained noise and were not engaged in the online sale of actual pharmaceuticals. On Instagram, we only detected 19 posts purportedly selling hydroxychloroquine and chloroquine, 2 posts selling remdesivir, and 1 post selling favipiravir. For Twitter, we detected 5 tweets selling hydroxychloroquine. All tweets selling hydroxychloroquine also concurrently sold PPE.

### COVID-19 Seller Metadata Characteristics

Sellers used key selling arguments common in e-commerce marketing that included offers of home delivery, free shipping, or discount codes to lower the price of COVID-19 testing kits and other products identified. Marketing tactics also included key selling argument terms such as “great news,” “flash sale,” “reliable,” “rapid,” “bulk sale,” and “immediate response” to give prospective buyers a sense of urgency and promote availability of products that were generally in scarcity in the legitimate supply chain during the study period. Other keywords included product descriptions that users could easily understand and identify including “immunity spray,” “Corona Kit,” and “IgM/IgG.” Because hashtags provide a way for users to curate topics of common interest, many posts included hashtags of the specific product they were selling (eg, #hydroxychloroquine, #IgM/IgG #test, and #testkit) in combination with general COVID-19 tags (eg, #coronavirus, #COVID9, and #rapidtest).

Generally, profiles of sellers included metadata and images that made them appear to originate from individual users. However, upon closer inspection, some of these accounts appeared to be cloned accounts with identical profile pictures and similar usernames that varied by only one or more characters to another more established and likely legitimate social media account. Accounts that were not represented as individuals or had affiliations were generally represented as medical supply or pharmaceutical companies. Individual and organizational accounts claimed to carry inventory of various COVID-19 testing kits and PPE, with alternative medicine–related accounts also selling various herbal remedies. Most posts contained pictures that included the product package, contents of the package and additional text, or had a general illustration of the SARS-CoV-2 virus. Some posts also included hyperlinks to external sites selling COVID-19 products, including 124 twitter posts (90 unique hyperlinks) and 41 Instagram posts (25 unique hyperlinks).

Contact information to enter into a transaction generally included instructions and details for direct messaging, WhatsApp numbers, email addresses, WeChat, and Skype for direct contact with seller. Some posts for testing kits also included hyperlinks to external e-commerce sites for purchase. Still other posts had descriptive text that linked to the user profile for additional contact information. A number of different languages were identified in the descriptive text of selling posts including English, Chinese, Japanese, Spanish, German, Arabic, Hindi, Russian, Ukrainian, Thai, and some others. For posts in a non-English language, coders self-translated those in Chinese, Japanese, Spanish, and Hindi, as coauthors spoke and read these languages. For other languages, the study team relied on Google Translate to assess the content of posts and if they were signals.

We noted that our deep learning classifier focuses on the detection of “selling” arguments (in the English language) and the presence of contact information from a seller. Hence, it is possible that not all non-English COVID-19 selling posts were detected, though non-English signal posts may contain the features subject to classification. The presence of non-English language posts and characters likely indicates that signal posts targeted non-US audiences and social media users, even though the majority of users on both of these platforms are located in the United States [29-31]. However, determining more precise geolocation of users was difficult as only 87 tweets and 134 Instagram posts had geotagged information available.

Generally, the metadata associated with the majority of signal posts indicated that there was medium to low levels of interaction with other social media users based on the number of likes, favorites, or retweets (the general metric of how much sharing and dissemination a post is getting on social media). The majority of tweets or Instagram posts had few likes, retweets, and followers. No signal posts were retweeted more than 50 times; 9 (0.86%) were retweeted more than 10 times, and 1033 (99.13% of unique tweets) were retweeted less than 10 times. For Instagram posts, the average number of “likes” for a signal post was 12.5, with 87 (15.5%) having more than 10 likes and 473 (84.4%) posts having less than 10 likes. For the interaction that was observed, we noticed that there was more interaction between sellers and other users in the comments section on Instagram compared to replies on Twitter. There were exceptions, with one detected twitter post from an account with over 97,000 Twitter followers and 1.5 million Instagram followers advertising sale of COVID-19 at-home finger stick IgG/IgM test on both Twitter and Instagram from what was characterized as a “LEGIT” supplier.

Although the majority of signal posts included contact information and instructions on purchasing the product, pricing information was included for less than 30 posts, primarily advertising sales of COVID-19 testing kits. The prices of testing kits ranged from US $4-$398 (all currencies converted to US dollars) for offers of individual kits as well as bulk orders. Individual kits were priced as low as US $4 to a maximum of US $375 with a mean cost of US $64.63 (SD $92.96) and a median cost of US $20.61/kit. Bulk kits were priced in the range of US $30.76-$398 for 25-50 kits/box with a mean cost of US $168.70 (SD $175.88). A questionable product described as a “portable hospital” device that claimed to use a negative ion current to treat COVID-19 and other viruses was priced at US $6000 (see Figure 3 for screenshots). Posts advertising availability of large quantities of testing kits also mentioned kits could be purchased at a cheaper price if ordered in bulk (hundreds to thousands). A few posts also included links to major e-commerce platforms such as eBay or AliExpress. Fiat currency was not limited to US dollars but included Euros, Pound Sterling, Indian Rupee, Philippine Peso, and other currencies. Additionally, payment transactions could be effectuated through PayPal or cryptocurrency such as Bitcoin.

## Discussion

### Principal Findings

This study used big data and machine learning approaches to detect and characterize illegal offers of sale for COVID-19 products on Twitter and Instagram. Overall, the total volume of illegal selling posts detected was low relative to the total volume of COVID-19 conversations collected (our nonfiltered general COVID-19 data set over this time period had over 165 million tweets and more than 272,000 Instagram posts), though the number of tweets and Instagram posts collectively were over 1000 representing a clear risk to patient safety. A possible reason for the small percentage of signal posts was that our data collection approach started with general COVID-19–related social media posts that were not specific to illegal sales but instead filtered for these terms after data collection was complete. As the overall volume of COVID-19 social media posts was extremely high, a more purposeful sampling approach focused on COVID-19–related health products or testing kits may have yielded a corpus with more signal.

Despite these limitations, we nevertheless identified over 1000 suspect selling posts, with the majority related to unapproved COVID-19 testing kits, which were detected at a time when access to legitimate COVID-19 testing in countries like the United States was extremely limited [32,33]. Based on the language, currency, and content of these posts, this infoveillance challenge also appears to be global, though the majority of posts detected were in the English language, reflecting the fact that most social media users on Twitter and Instagram are located in the United States. Far fewer posts were detected for therapeutic products, though separate research conducted by our own group and others have turned up various drugs (including hydroxychloroquine, chloroquine, and favipiravir), vaccines, and even blood plasma offered for sale via illegal online pharmacies, e-commerce sites, and on the dark web [34,35].

The lack of signal posts for COVID-19 therapeutics may indicate that product segmentation on different parts of the internet is occurring. Specifically, illegal online pharmacies and dark web marketplaces may have already been selling these products outside of the context of treating COVID-19, as many of these drugs are already approved for other indications, diminishing the opportunity or need for direct sales to consumers via social media. Consumer demand for drugs may have also been muted as the number of confirmed COVID-19 cases at the time was relatively low and there was limited evidence regarding the efficacy of these products or their given active pharmaceutical ingredient to treat COVID-19. Instead, a widespread lack of access to testing may have made getting a COVID-19 diagnosis a priority before seeking treatment, reflected in our high detection of suspect COVID-19 testing kits.

Some posts detected by our data collection process had already been taken down from the platforms at the time of manual inspection, indicating that platforms may have been self-policing and removing this content given that it violates their existing terms of use or specific content moderation policies related to COVID-19 [36,37]. In fact, many social media platforms indicate they are attempting to address the concern of both COVID-19–related misinformation and cybercrime [38,39]. Yet at present, it is difficult to assess the effectiveness of platform self-regulation. Despite detecting that certain signal posts had been removed (such as those with obvious coronavirus-related account names or descriptions), other detected posts nevertheless remained active and accessible to users after our study was completed, evidencing that more work needs to be done to reign in this part of the COVID-19 infodemic.

Importantly, the presence of this type of criminal activity and fraud on social media is not a new phenomenon, as cybercriminals are keen to take advantage of the anonymity, convenience, and accessibility to the public that these platforms offer to advance crimes of opportunity. In the case of COVID-19, we are arguably in the midst of a “cyber syndemic,” where the public health consequences of COVID-19 simultaneously interact with the unique risks associated with the internet and social media together, which can worsen the spread of the disease. Specifically, the posts detected in this study can bring both economic and health harm by introducing unproven, substandard, falsified, and counterfeit health products to those afflicted by COVID-19, leading to financial loss while also increasing the risk of disease spread by negatively influencing health behaviors [40].

Reflecting the real-world consequences of COVID-19–related crimes, the US Federal Trade Commission estimates that there have already been US $40 million in losses due to COVID-19 fraud [41]. Law enforcement groups such as the US Customs and Border Protection have intercepted hundreds of fake COVID-19–related products at borders and have launched several initiatives such as Homeland Security Investigations’ “Operation Stolen Promise” and the S.T.O.P COVID-19 Fraud Campaign [42]. The US Federal Bureau of Investigation reported a 300% increase in fraud and cybercrime scams since 2019-nCoV appeared [43]. Operation Pangea, an Interpol-led takedown of illicit internet sites, focused its March 2020 activities on COVID-19 scams [44]. It found extensive and growing fraud for coronavirus medical “treatments,” cures, and protective equipment as well as, more recently, sales of all forms of chloroquine [45]. The European Anti-Fraud Office also announced that the European Union will dedicate resources to target fake coronavirus medical and protection products being sold online.

However, combatting COVID-19 cybercrime, and more specifically illegal online sales of COVID-19 health products, is a dynamic challenge. Existing difficulties of interdicting the global illegal online trafficking of counterfeit and falsified products are accelerated and accentuated during a pandemic, as information rapidly changes, misinformation proliferates, and platforms struggle to self-regulate large and diverse volumes of content on the pandemic. In contrast, black markets can be adaptive to these types of crises, with scammers seeking to take advantage of confusion and heightened concerns about safety and health risks to target vulnerable consumers with fake products and treatments [46].

To address these challenges, a data-driven public health intelligence approach is needed. Specifically, although the results of this study are informative to the characteristics of illegal COVID-19 online sales during early stages of the outbreak, they are nevertheless static, only reflecting the degree of risk to the public at a single point-of-time. Instead, active surveillance of illegal COVID-19 digital marketplaces is needed, along with the use of visualization tools that can provide needed data intelligence to understand the constantly changing dynamics of this infodemic threat. Recognizing this need, we have developed a prototype data dashboard on the open source platform Redash that can be used by public health officials, drug regulatory authorities, and law enforcement agencies that visualizes our ongoing big data digital surveillance work to detect and classify illegal marketing, sales, and trafficking of COVID-19–related products on social media platforms and other parts of the internet. The public version of the dashboard can be viewed at [47]. The dashboard reports and visualizes characteristics of illegal selling posts including location (if available), generates a list of top-related hashtags, captures images of suspect products, and analyzes other metadata about selling activity. We have shared a version of this dashboard with colleagues at the WHO and FDA in hopes that it can help improve and accelerate content removal, increase awareness of the risks to consumers, and lead to a safer online environment in the midst of this ongoing pandemic.

### Limitations

Our study has certain limitations. First, it was limited to a relatively short period of data collection, which limits the generalizability of the results to overall illegal COVID-19 social media–based offers for sale. Our corpus of social media posts included general COVID-19 keywords and filtered terms, but it is possible that some COVID-19 sellers do not include these terms in their posts. Hence, we may have failed to collect posts with suspect COVID-19 sales. We did not engage sellers or other social media users to verify if COVID-19 products were actually available, so we cannot say with certainty that advertised products were being sold, whether they were economic frauds and scams, or if they were products approved outside the United States. We relied on textual analysis to identify selling posts and did not use multimodal approaches that could analyze and classify both text and images, a method that could potentially improve classification [48-51]. Finally, due to the time lag in collecting social media messages, conducting our topic modeling, and then classifying posts using our machine learning inference phase, some posts were no longer available for manual inspection as they had been removed from platforms, so we could not further validate their content. These posts may have been self-deleted posts or removed as they violated the terms of use for these platforms.

### Conclusion

Our study provides a snapshot of the characteristics of illegal online sales of COVID-19–related health products on two popular social media platforms: Twitter and Instagram. It also details an innovative methodology using a combination of unsupervised and supervised machine learning to detect illegal sales during a global pandemic. Unfortunately, illegal online sales of COVID-19 health products are likely to continue and possibly accelerate as this health emergency continues to progress. A “flattening of the curve” will not halt the progression of this parallel infodemic, as the public continues to desperately seek access to COVID-19 testing, therapeutics, and an eventual vaccine. As legitimate news about promising and new COVID-19 treatments and countermeasures becomes available, scammers and counterfeiters will inevitably seek to capitalize on desperation and high demand from global citizens who simply want to be safe and prepared against this historic disease. Future studies should continue to explore the dynamic nature of the COVID-19 cyber syndemic and build solutions to prevent its digital spread.

## Appendix

Multimedia Appendix 1 Additional details regarding study methods.

Multimedia Appendix 2 Coding scheme for social media posts.

## Abbreviations

API: application programming interface
BTM: biterm topic model
COVID-19: coronavirus disease
e-commerce: electronic commerce
FDA: Food and Drug Administration
NLP: natural language processing
PPE: personal protective equipment
SARS-CoV-2: severe acute respiratory syndrome coronavirus 2
WHO: World Health Organization
2019-nCoV: novel coronavirus
