# Supplementary material for: Big Data, Natural Language Processing, and Deep Learning to Detect and Characterize Illicit COVID-19 Product Sales: Infoveillance Study on Twitter and Instagram
Source: JMIR Public Health Surveill. 2020 Aug 25;6(3):e20794. doi: 10.2196/20794 (PMC7451110; doi:10.2196/20794)
Supplement: Multimedia Appendix 1 [file publichealth_v6i3e20794_app1.docx]

**Supplementary File 1: Additional Details Regarding Study Methods**

**Data collection period timeline:**

This study utilized the Twitter public API stream for collecting COVID-19-related posts on Twitter (from March 3^rd^ to April 11^th^ 2020) and a customized web scraper developed in the computer programming language Python™ to collect COVID-19-related posts on Instagram (from February 5^th^ to May 7^th^). Using this approach, we were able to collect both prospective (Twitter) and prospective and retrospective data (Instagram). Search parameters included posts source, post type, tagged location, and date posted. General COVID-19 terms used for this study included:

*General COVID-19 keywords: “covid19”,“corona”,“coronavirus”,“wuhan” “coronavid19”,"outbreak",“covid",and "pneumonia"*

The following filtered terms related to COVID-19 suspected health products were used based on manual searches conducted prior to the commencement of this study. These filtered terms were also chosen based on news and events that emerged during the study timeframe about COVID-19 cures, testing, and pharmaceutical treatments. The full list of filtered keywords for each category of COVID-19 health products is available below:

*Fake cures/Herbal Products terms (Wave 1): 'Superblue Toothpaste', 'Anti-coronavirus', 'Tea',*

*'Essential oils', 'Tinctures', 'Herbal extracts', 'Vinegar', 'Silver', 'Colloidal silver',*

*'Vitamins', 'Heritage seeds', 'Seeds', 'Naturopathic', 'Holistic', 'Aromatherapy', 'Vitamin C', 'Herbal', 'Alcohol', 'Ethanol', 'Methanol', 'Chlorine', 'Lozenges', 'Garlic', 'Salt water', 'Mineral', 'Supplement', 'Antiviral', 'HIV', 'Cocaine', 'Xephyr' ,'Potion', 'Pill', 'Tonic', 'Dietary', and 'Vital Silver'*

*COVID-19 testing kits terms (Wave 2): "rapid", "testing", "kit", "antibody", "fingerstick", 'IgG', 'IgM', 'rapidtest', '#testcovid19', '#rapidtestcovid', '#coronatest', '#rapidtestcovid19'*

*Pharmaceuticals terms: "Hydroxychloroquine", "Remdesivir", "Favipiravir", "Kaletra", "Aluvia", "chloroquine", and "convalescent plasma"*

**Data Processing, Analysis and Classification:**

*Unsupervised Topic Modeling:* Using unsupervised NLP approaches can help discover underlying patterns in data quickly. For example, given a large text corpus, a topic model can succinctly summarize major topics (e.g. illegal COVID-19 sales posts versus news topics related to COVID-19 treatment) by output of a certain predetermined number of groups of words highly correlated to each other [21]. Using BTM, groups of messages/text containing the same word-related themes are categorized into clusters, the main themes of those clusters is considered as the topic of the aggregation of the text [21].  We used BTM to extract clusters of social media conversation that we believed contained signal posts. Each of the thematic groups output by BTM were then marked as to whether they were relevant by assessing whether the frequency, distribution, and combination of word groupings in output topics included key “selling argument” topics (e.g. “contact me”, “price”, “sale”, “available”, etc.) [25].  After “signal” topic groups were isolated, Twitter and Instagram posts were retrieved and manually annotated by authors in order to confirm if posts in these topics were offering the sale of COVID-19 products. This labelled dataset was then used for our machine learning inference phase.

*BTM K-value Selection:* We set a total number of k different clusters for filtered data, resulting in texts with similar themes put into the same cluster, if the k value is too large, the text with the same theme could be separated into two clusters, if the k value is too small, the topic(s) with weak signals (i.e. small volume of discussion) could be obscured by topic(s) with stronger signals (i.e. larger number of discussions). To find the appropriate k value for this data set, we use the u-mass coherence score to determine the k value. Coherence score is used to measure the performance of a topic model with different number of clusters, it can help distinguish between topics that are semantically interpretable and topics that are artifacts of statistical inference. A k value with higher coherence score means the clusters it categorizes are more identical to each other. We let D(v) be the document frequency of the word type v (i.e., the number of documents containing at least one token of type v) and D(v, v0) be the co-document frequency of word types v and v0 (i.e., the number of documents containing one or more tokens of type v and at least one token of type v0). We define topic coherence as:

C(t;$v^{t}$) = $\sum_{m=2}^{M} \sum_{l=1}^{m-1} log(\frac{D\left( v_{t}^{m},v_{t}^{l} \right)+1}{D(v_{l}^{t})})$

where V(t) = ${(v}_{t}^{1}{\ldots,v}_{t}^{m}$) is a list of the M most probable words in topic t.

We then choose 8 different k values (i.e 5,10,15,20,25,30,35,40) for the BTM. We select the k values with highest coherence score, the theme from these k different cluster should be most distinguishable so we can avoid missing a topic with low volume of data.

*BTM Results and Limitations:* For each group of keywords, after running BTM to identify topic clusters likely associated with signal posts and then manually labelling them, we detected 524 signal tweets and 265 signal Instagram posts. This low volume of signal posts generally suggests that BTM may not be a suitable model for accurately detecting COVID-19 product illegal selling in large volumes of data, hence, necessitating supervised machine learning approaches that can adapt existing models to detect illegal health product selling to COVID-19-related seller and product features. The reasons for these limitations include that BTM is more efficient at detecting topic clusters that have enough volume to be isolated as distinct topic clusters in comparison to topics with lower volumes of messages, which also depends on the k-value of topics outputted by the model. If the topic has a low volume compared to other topics (i.e. the total volume of topics related to illegal COVID-19 product sales being much smaller than general conversations or news reports about COVID-19 experiences with testing and concerns about treatment), then these lower volume topics may be harder to detect in the entire corpus. It is for this reason; we chose to filter data for terms we believed were associated with COVID-19 suspect product sales prior to running BTM. However, even within this filtered dataset, actual sales of COVID-19 health products may have been small relative to the general conversations, news mentions, and regulatory and public service announcements associated with these terms. This necessitated using a combination of a low volume of training data with an existing model already efficient at detecting illegal health product sales.

*Machine Learning Model Development:* Since the volume of signal results retrieved from BTM were not sufficient to train a new model, we used a previous deep learning model that we developed to detect illegal sale of controlled substances and other illicit drugs as our pretrained model, which was adopted for COVID-19-related signal [26].  Details of this model, which uses deep learning using a recurrent neural network with long short-term memory (LSTM) unit to study the text and syntax of social media posts is available in a previous published study in JMIR [26].  The strengths of this approach are that is allows us to adapt an existing model to a similar profile of messages for a different product feature (i.e. COVID-19 products versus drugs). The possible challenges associated with this approach are the lack of precision in detecting COVID-19 sellers that do not include contact information or “selling” arguments in their social media posts or have selling posts that are fundamentally different from the way drug sellers approach their sales and marketing tactics. We addressed this limitation by conducting manual searches to first identify COVID-19 sellers and determine if they were similar to past observations with drug dealers. Our initial observations indicated that they used similar selling arguments and sales tactics, hence, the model was adopted accordingly.  However, it is possible that certain COVID-19 sellers may have used different or changed their tactics to selling in response to having their posts taken down or in anticipation of the unique consumer behavior dynamics of the COVID-19 outbreak itself.

**Figure A** shows the deep learning model adapted and developed for this study compared to the model previously developed for illegal drug selling detection. Before we input the text into the pretrained model, each text is transformed into a vector with a dimension (60, size of dictionary).  These comprise the signals contained in one text, and then the model converts these signal layers by layering a more specific feature vector with a smaller size.  We then extract the vector from the Dense Layer 3 as the feature vector filtered by the pretrained model for each text, the feature from this layer should contain the features of the online seller. Since the feature we extract correspond to the drug sellers in our previous study, we use the same text block as our preexisting illegal drug selling model to extract the features from the text-only vector. Since this dataset contains both Twitter and Instagram data, we observed that our tweets had fewer hashtags compared to Instagram posts.  For this reason, we replace the Hashtag Block with the pre-trained model which will output the feature vector with the same dimension.

**Figure A:** Comparison of Deep Learning Model for Detecting Illegal Drug Dealing and Adapted COVID-19 Illegal Selling Model Used in this Study

Image A: Deep Learning model for drug-dealing model


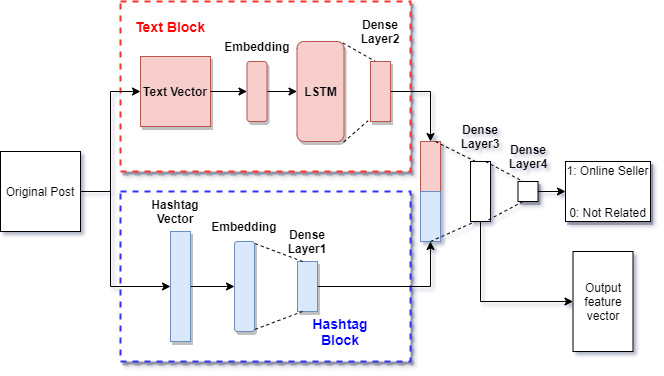


Image B: Deep learning model for COVID-19 selling text detection


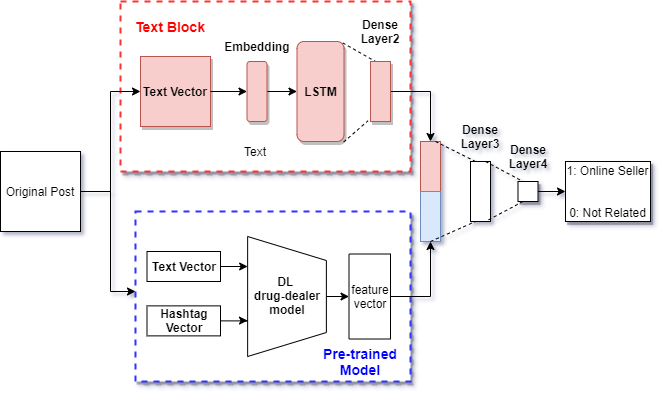


**Model Parameters:** For this study, the parameters of the model included setting the length of the text vector to 60 words, which is the average length of texts in our COVID-19 dataset. If the text was longer than 60 words, we only chose the first 60 words to maintain the efficiency of the model; if it was shorter, we filled the rest of the vector with 0. Then we used word embedding to generate the text into a word vector with dimensions (60, 400). The strategy for Instagram hashtags was the same, but the average length of hashtags was set to 15 characters.

**Note: Embedding Layer:  input_dim is based on the size of the dictionary for each phase of data, the input_dim for Text is 60, input_dim for Hashtag is 15, the output_length is 400.**

**LSTM layer:** contain 800 units, with dropout = 0.2, recurrent_dropout = 0.2

Dense Layer 1: unit = 200, activation = sigmoid

Dense Layer 2: unit = 200, activation = sigmoid

Dense Layer 3: unit = 200, activation = sigmoid

Dense Layer 4: unit = 1, activation = sigmoid

Optimizer: Adam

Loss: Binary_crossentropy

*Model Evaluation:* We used the labelled data from BTM (which included the true positive signal tweets and false positive signal tweets) as our training set, which contained a total of 2676 tweets and 1471 Instagram posts. We then separated the training set and validation set in a 3:7 proportion, with model evaluation statistics provided in **Table 1**. We compared our current model with the previous model that was used for detecting online drug selling and found that the current COVID-19 model had better performance on Instagram than Twitter, but also had higher precision in distinguishing signal tweets and non-signal tweets when compared to classification of Instagram posts.

***Table 1.*** *Deep Learning* *Model Evaluation and Performance*

|  | | *Precision* | *Recall* | *F1 score* |
| --- | --- | --- | --- | --- |
| *Instagram* | *Drug Model* | *0.628* | *0.445* | *0.520* |
|  | *Covid Model* | *0.780* | *0.664* | *0.717* |
| *Twitter* | *Drug Model* | *0.726* | *0.356* | *0.478* |
|  | *Covid Model* | *0.811* | *0.457* | *0.585* |

**Study “Signal” Data Availability:**

Data that was labelled as signal for the purposes of this study are anonymized and available based on their Twitter ID via author’s GitHub repository at: <https://github.com/Mathison/Coronavirus_product_social-media_post_id>
