# Supplementary material for: Big Data, Natural Language Processing, and Deep Learning to Detect and Characterize Illicit COVID-19 Product Sales: Infoveillance Study on Twitter and Instagram
Source: JMIR Public Health Surveill. 2020 Aug 25;6(3):e20794. doi: 10.2196/20794 (PMC7451110; doi:10.2196/20794)
Supplement: Multimedia Appendix 2 [file publichealth_v6i3e20794_app2.docx]

**Supplemental File 2: Coding Scheme for Social Media Posts**

Manual annotation of Tweets and Instagram Posts: The following coding scheme was used for binary classification of whether the tweet or Instagram posts advertised sale of a COVID-19 health product and if a post offered specific COVID-19 product/s for sale and if a contact or purchase method was made available.

| **THEME** | **SIGNAL** | **CODING SCHEME** |
| --- | --- | --- |
| **Infodemic Wave 1**  (Fake cures/Herbal products) | YES | - Tweets advertising sales of immunity boosting products, herbal products/ concoctions as alleged cure/prevention for COVID-19 - Tweets promoting “DIY” instructions on how to prepare herbal concoctions that can cure COVID-19 - Advertising sales of food supplements with an alleged tag of COVID-19 cure/prevention |
|  | NO | - Tweeting news posts on spread of misinformation on COVID-19 cure - Users discussing possible immunity boosting remedies in news/social media without advertising sales or promotion as cure/prevention for COVID-19 - Retweeting legitimate warnings from public health, law enforcement and other sources - Tweeting news alerts - Sarcasm/jokes on cures for COVID-19 |
| **Infodemic Wave 2**  (Testing kits, PPE) | YES | - Tweets advertising sales of unapproved COVID-19 testing kits (IgG, IgM, rapid test kits) and mention of DTC (direct-to-customer) - Tweets advertising sales of “at-home” or “DIY” testing kits - Tweets advertising sales of one or more PPE (masks, gloves, protective suits etc.,) - Pictures of the testing kits/PPE with contact information and pricing details |
|  | NO | - Tweets about testing sites/ testing availability - User discussions on rapid testing kits under research - Tweets on scarcity of masks, gloves, other protective gear - User discussions about testing kits without actual advertising of sales/pricing information - Retweeting news alerts on testing/PPE scarcity - Tweeting about the mention of “lack of testing/PPE” by politicians/health officials/government officials - Sarcasm/jokes on COVID-19 testing/PPE |
| **Infodemic Wave 3**  (Pharmaceuticals) | YES | - Tweets advertising sale of therapeutics/ pharmaceutical drugs such as Hydroxychloroquine, Remdesivir, Favipiravir and other drugs under evaluation by drug regulatory agencies. - Pictures of the drug packets with contact information and pricing details |
|  | NO | - User discussions on potential candidate drugs that are under research for use in COVID-19 patients - User discussions about treatment options available in different countries - Tweeting about the possibility of the above-mentioned drugs to treat COVID-19 - Tweets about clinical trials for these drugs - Retweeting legitimate warnings from public health, law enforcement and other sources - Tweeting news alerts - Sarcasm/jokes |
